# The Active Sulfate-Reducing Microbial Community in Littoral Sediment of Oligotrophic Lake Constance

**DOI:** 10.3389/fmicb.2019.00247

**Published:** 2019-02-13

**Authors:** Susanne Wörner, Michael Pester

**Affiliations:** ^1^Department of Biology, University of Konstanz, Konstanz, Germany; ^2^Leibniz Institute DSMZ – German Collection of Microorganisms and Cell cultures, Braunschweig, Germany; ^3^Institute for Microbiology, Technical University of Braunschweig, Braunschweig, Germany

**Keywords:** lake sediment, next-generation amplicon sequencing, chitin, sulfate reduction, dsrB transcripts

## Abstract

Active sulfate-reducing microorganisms (SRM) in freshwater sediments are under-examined, despite the well-documented cryptic sulfur cycle occurring in these low-sulfate habitats. In Lake Constance sediment, sulfate reduction rates of up to 1,800 nmol cm^-3^ day^-1^ were previously measured. To characterize its SRM community, we used a tripartite amplicon sequencing approach based on 16S rRNA genes, 16S rRNA, and *dsrB* transcripts (encoding the beta subunit of dissimilatory sulfite reductase). We followed the respective amplicon dynamics in four anoxic microcosm setups supplemented either with (i) chitin and sulfate, (ii) sulfate only, (iii) chitin only, or (iv) no amendment. Chitin was used as a general substrate for the whole carbon degradation chain. Sulfate turnover in sulfate-supplemented microcosms ranged from 38 to 955 nmol day^-1^ (g sediment f. wt.)^-1^ and was paralleled by a decrease of 90–100% in methanogenesis as compared to the respective methanogenic controls. In the initial sediment, relative abundances of recognized SRM lineages accounted for 3.1 and 4.4% of all bacterial 16S rRNA gene and 16S rRNA sequences, respectively. When normalized against the 1.4 × 10^8^ total prokaryotic 16S rRNA gene copies as determined by qPCR and taking multiple rrn operons per genome into account, this resulted in approximately 10^5^–10^6^ SRM cells (g sediment f. wt.)^-1^. The three amplicon approaches jointly identified *Desulfobacteraceae* and *Syntrophobacteraceae* as the numerically dominant and transcriptionally most active SRM in the initial sediment. This was corroborated in the time course analyses of sulfate-consuming sediment microcosms irrespective of chitin amendment. Uncultured *dsrAB* family-level lineages constituted in sum only 1.9% of all *dsrB* transcripts, with uncultured lineage 5 and 6 being transcriptionally most active. Our study is the first holistic molecular approach to quantify and characterize active SRM including uncultured *dsrAB* lineages not only in Lake Constance but for lake sediments in general.

## Introduction

Lake Constance is a typical pre-alpine lake that has currently an oligotrophic status and is oxygenated down to the sediment (Güde and Straile, 2016). Within the first millimeters of its sediment, oxygen is completely depleted (Gerhardt et al., 2005) leaving room for anaerobic degradation of organic matter either by fermenters that share their degradation products (acetate and H_2_) with methanogenic archaea (Rothfuss and Conrad, 1993; Thebrath et al., 1993) or by nitrate- (Hauck et al., 2001), Fe (III)- (Gerhardt et al., 2005), humic acid- (Kappler et al., 2004), and sulfate-reducing microorganisms (Bak and Pfennig, 1991a,b). A major component of organic matter entering the sediment is chitin as an abundant biopolymer in aquatic environments (Gooday, 1990; Cauchie, 2002). It consists of linked amino-sugar subunits with ß-1,4-N-acetyl-D-glucosamine as the monomeric structure and serves as structural element in the exoskeleton of arthropods (e.g., zooplankton) and the cell wall of fungi and certain algae and protozoa (Gooday, 1990). The estimated annual production of 28 × 10^6^ tons by crustaceans and insects in freshwater ecosystems and its efficient turnover in freshwater sediments (50–75%, Beier and Bertilsson, 2013) indicate chitin as an important carbon and nitrogen source for microorganisms in these habitats (Gooday, 1990; Cauchie, 2002).

In Lake Constance, sulfate concentrations of 50–300 μM can be measured in the upper layers of the littoral sediments. Here, maximum sulfate reduction rates (SRR) as determined using ^35^S-SO_4_^2-^ as a radiotracer reached values of 1,800 nmol cm^-3^ day^-1^ at 1–2 cm sediment depth (Bak and Pfennig, 1991a). This surpasses typical SRR in sulfate-rich marine surface sediments by one order of magnitude, where sulfate reduction is one of the major anaerobic carbon degradation pathways (Jørgensen, 1982; Bowles et al., 2014). The high SRR at concomitant low sulfate concentrations in Lake Constance are explained by a fast and effective cycling of sulfur species between their oxidized and reduced states, which is controlled by a cryptic sulfur cycle as is typical for freshwater sediments and wetlands (Pester et al., 2012). In particular, sulfide as the end product of sulfate reduction does not accumulate beyond 80 μM in littoral sediments of Lake Constance (Gerhardt, 2004), but is likely rapidly re-oxidized back to sulfate at oxic-anoxic interfaces or under completely anoxic conditions using nitrate, Fe (III), or humic acids as electron acceptor (Pester et al., 2012). SRR in Lake Constance sediments are saturated at 60 μM sulfate, reaching maximum values of 3,000 nmol cm^-3^ day^-1^ in sediment slurries (Bak and Pfennig, 1991a). This shows that SRR are not limited by the prevailing sulfate concentrations in Lake Constance (Bak and Pfennig, 1991a; Güde and Straile, 2016), which is also true for many other freshwater lakes (Holmer and Storkholm, 2001).

To date, SRM are known to belong to the five bacterial lineages *Deltaproteobacteria*, *Nitrospirae*, *Clostridia*, *Thermodesulfobiaceae*, and *Thermodesulfobacteria* as well as the two archaeal lineages *Euryarchaeota* and *Crenarchaeota* (Muyzer and Stams, 2008). Using metagenomics-guided discovery of novel microorganisms, three recent studies revealed the capacity for dissimilatory sulfate or sulfite reduction in at least 13 additional bacterial and archaeal lineages, which were so far not associated with this metabolic trait (Anantharaman et al., 2018; Hausmann et al., 2018; Zecchin et al., 2018). These included among others the *Acidobacteria*, *Planctomycetes*, *Verrucomicrobia*, and *Armatimonadetes*. In specific, 8 of these 13 newly identified SRM groups are candidate phyla without isolated representatives (Anantharaman et al., 2018). In parallel, microbial diversity assays based on the functional marker genes *dsrAB*, which encode the dissimilatory sulfite reductase and are commonly used as diagnostic markers in detecting SRM, indicate an even larger phylogenetic diversity with at least 13 uncultured family-level *dsrAB* lineages (Pester et al., 2012; Müller et al., 2015). So far, the latter only partially overlap with the metagenome-discovered putative SRM, e.g., uncultured *dsrAB* lineage 8 is represented by *Acidobacteria* (Hausmann et al., 2018) and uncultured *dsrAB* lineage 13 is closely related to mesophilic *Nitrospirae* (Zecchin et al., 2018). Extending upon the phylogenetic framework for *dsrAB* genes that was proposed by Müller et al. (2015), a recent *dsrB* amplicon survey of 200 environmental samples including various freshwater habitats increased the number of known species-level operational taxonomic units (OTUs) related to potential SRM from ca. 800 (Müller et al., 2015) to more than 150,000 (Vigneron et al., 2018). Considering the ca. 240 cultivated sulfate-reducing microbial species (Rabus et al., 2013), this result uncovered that less than 0.2% of all potential SRM are available in pure culture. The same study revealed that among uncultured SRM those belonging to uncultured *dsrAB* lineage 5 are quite prevalent in freshwater habitats (Vigneron et al., 2018).

Despite the importance of sulfate reduction for the sediment biogeochemistry of Lake Constance as a typical freshwater lake, its SRM community is so far very poorly characterized. The only attempt was based on enrichment cultures grown on H_2_, lactate, acetate, propionate or long chain fatty acids, with isolates being affiliated to the genera *Desulfovibrio*, *Desulfobulbus*, and *Desulfotomaculum* (Bak and Pfennig, 1991b). Therefore, we attempted to systematically analyze and quantify the littoral SRM community of Lake Constance using a tripartite, high throughput amplicon sequencing approach based on 16S rRNA genes, 16S rRNA, and *dsrB* transcripts. To delineate active SRM, sediment was incubated in the presence of chitin as a general substrate for the complete anaerobic degradation network in the presence and absence of externally supplied pulses of sulfate. This was compared to sediment incubated with sulfate pulses only or without any external additions. Our results show that SRM constitute about 3% of the total bacterial sediment community with their major representatives being affiliated to the deltaproteobacterial families *Desulfobacteraceae* and *Syntrophobacteraceae*.

## Materials and Methods

### Experimental Set Up

Sediment was taken from the littoral area of the Mainau Bay (Obere Güll) in Lake Constance (N47°42′7′′ E9°11′43′′), Germany on September 15, 2015. Three sediment push cores were sampled at 2 m water depth with plastic tubes of 80 mm inner diameter. At this shallow water depth, the *in situ* temperature of the sediment is strongly influenced by the temperature of the overlaying water column and can range from 4°C in winter (Bak and Pfennig, 1991a) to 23°C in summer (Bak, 1988). The sediment structure and layering were preserved in the cores, spanning a depth of 0–30 cm. Cores were covered with lake water and brought to the laboratory. On the same day, the first centimeter was sliced off by a sterile metal plate from each core to remove the oxygenated layer. The following two centimeters (1–3 cm below surface) were then removed in one piece and immediately placed in a sterile anoxic tank, where the sediments of these cores were homogenized and divided into 12 sterile 150-mL glass bottles by 50 g portions under a constant flow of 100% N_2_. 40 ml of anoxic, filter-sterilized (0.2 μm) surface lake water was added to the sediment. All microcosms were sampled for their respective initial sediment under a constant flow of 100% N_2_, sealed with butyl-rubber stoppers, and pre-incubated for seven days at 15°C to deplete internal substrates within the sediment. Thereafter, microcosms were split in triplicates into four incubation lines and further incubated at 15°C. Every 3–4 days, we amended sulfate at dosages of 711 ± 539 μM to six of the microcosms. Three of these received once 150 mg chitin (Sigma-Aldrich, Darmstadt, Germany) as a general and abundant organic carbon source in aquatic sediments (incubation line 1: “chitin & sulfate”) as compared to sulfate-stimulated control (incubation line 3: “sulfate only”). To control in parallel for community members that are active in the methanogenic degradation network, the other six microcosms were either incubated just with chitin (incubation line 2: “chitin only”) or without any addition (incubation line 4: “control”) (Supplementary Figure S1).

### Chemical Analyses

Sulfate and degradation products of chitin were monitored every time incubation lines 1 and 3 were supplemented with sulfate. Gas samples from the headspace (200 μl) were monitored for accumulation of CH_4_ and CO_2_ by gas chromatography (6000 Vega Series 2 GC, Carlo Erba, Italy), using a 45/60 carboxen 1000 column (Supelco, Oberhaching, Germany) operated at 120°C and a thermal conductivity detector. 100% nitrogen gas was used as carrier gas at a column pressure of 60 kPa. The injection port and detector were heated to 150 and 180°C, respectively. Liquid samples (500 μl) were periodically taken either once for incubation lines without sulfate supplementation (lines 2, 4) or before and after sulfate addition (lines 1, 3). These samples were centrifuged (4°C, 14,000 ×*g*, 5 min) and the supernatant was stored at –20°C until analysis. Before measurements, samples were centrifuged again (4°C, 14,000 ×*g*, 5 min) and the supernatant was used for analysis. Sulfate concentrations were determined photometrically by a barium chloride turbidity test (Tabatabai, 1974). Formate, acetate, propionate, butyrate, and lactate were monitored by HPLC (Shimadzu, Munich, Germany) equipped with an Aminex HPX87H column (BioRad, Munich, Germany) heated to 45°C with 10 mM H_3_PO_4_ as eluent at a flow rate of 1 ml min^-1^. Analytes were detected with a photo diode array detector at 200 nm (Shimadzu). The detection limit of all analyzed compounds was 10 μM.

### Sediment Sampling and Total Nucleic Acids Extraction

Sediment in the microcosms was sampled at four different time points (Supplementary Figure S1). First samples were taken right at the onset of the experiment and equaled the initial sediment; all other samples were collected after 9, 21, and 43 days of incubation, which were preceded by 7 days of pre-incubation. Sediment samples were frozen immediately in liquid N_2_ and stored at -60°C until further processing. RNA and DNA were extracted from the same samples using the RNA PowerSoil^®^ Total RNA Isolation Kit in combination with the RNA PowerSoil^®^ DNA Elution Accessory Kit (Mo Bio Laboratories Inc., Carlsbad, CA, United States). Residual DNA in the RNA was removed with the TURBO DNA-free Kit (Ambion, Thermo Fisher Scientific, Darmstadt, Germany). Transcription of RNA into cDNA was performed using SuperScriptIII (Life Technologies, Darmstadt, Germany). To remove traces of remaining RNA, extracted DNA was treated with RNase ONE (Promega, Mannheim, Germany). RNA and DNA were quantified using Ribo- and PicoGreen (Life Technologies), respectively.

### Quantification of 16S rRNA Gene Copies

Quantitative PCR (qPCR) of total bacterial and archaeal 16S rRNA genes was performed on an ABI 7500 cycler (Applied Biosystems) with the primer pair 1389F (5′-TGY ACA CAC CGC CCG T-3′) and 1492R (5′-GGY TAC CTT GTT ACG ACT T-3’) as described in detail previously (Hausmann et al., 2016). Besides an obligatory melting curve analysis, selected qPCR products were visualized by agarose gel electrophoresis to verify absence of unspecific PCR products. Amplification efficiencies had an average of 95 ± 4%. For qPCR assays a standard curve with a purified 16S rRNA gene PCR product of an *Acetobacteroides* clone was generated from 5 × 10^1^ to 5 × 10^7^ template copies per assay (*R*^2^ = 0.99). Absence of PCR-inhibitory substances was confirmed by qPCR analyses of dilution series of two selected sediment DNA extracts of this study.

### Amplicon Sequencing of 16S rRNA Genes and cDNA

The V3–V4 region of bacterial 16S rRNA genes was amplified using the universal primer set 341F (5′-CCT ACG GGN GGC WGC AG-3′) and 805R (5′-GAC TAC HVG GGT ATC TAA TCC-3′) (Herlemann et al., 2011). PCR amplification from DNA extracts and cDNA, barcoding and Illumina amplicon sequencing was performed at Microsynth (Baldach, Switzerland). Initial PCR amplification was performed by an annealing temperature of 56°C using 20 cycles. In a second PCR, barcodes were added to the initial amplicons using 10 cycles (DNA) or 8 cycles (cDNA) at an annealing temperature of 56°C.

Amplicon reads were subjected to quality control and *de novo* chimera filtering (UCHIME, Edgar et al., 2011) as implemented in Mothur v. 1.38.1 (Schloss, 2009). 5,362,183 high-quality reads remained and formed 408,146 species-level OTUs (97% sequence identity). Taxonomic identity was assigned with the RDP Classifier (Wang et al., 2007) and the RDP 16S rRNA training set 16 using a confidence threshold of 0.8.

### Illumina Sequencing of dsrB cDNA

RNA extracts were reverse transcribed and subsequently amplified using a one-step RT-PCR system (Access RT-PCR System, Promega). PCR amplification of *dsrB*-fragments (∼400 bp) was performed according to a modified protocol described by Pelikan et al. (2016). The utilized primers were DSR1728F mix (5′-CAY ACC CAG GGN TGG-3′) and DSR4R-mix (5′-GTR WAR CAR TTD CCR CA-3′) (Steger et al., 2011) elongated by standard M13f (5′-CAG GAA ACA GCT ATG AC-3′) and M13r (5′-GTA AAA CGA CGG CCA G-3′) primers, respectively. 75 ng RNA was reverse transcribed for 45 min at 45°C using only the reverse primer. After cDNA synthesis, the forward primer was added and the cDNA was initially denatured for 2 min at 94°C followed by 30 cycles of denaturation (95°C, 30 s), primer annealing (55°C, 30 s), elongation (72°C, 30 s) and a final extension at 72°C for 7 min. PCR products were purified by magnetic beads (Agencourt^®^ AmpureXP, Beckman Coulter, Brea, United States). In a second PCR, 1 μl of the purified PCR product was used as template targeted by M13-primers elongated at each side with a 6-bp long barcode. Barcodes differed from each other by at least two nucleotides. The M13 PCR was performed using 15 cycles of denaturation (95°C, 30 s), primer annealing (60°C, 30 s), elongation (72°C, 40 s) and a final extension at 72°C for 5 min. PCR products of correct length were purified with the MiniElute Gelextraction kit (Qiagen, Hilden, Germany), quantified by PicoGreen, and mixed in equal amounts for sequencing on an Illumina MiSeq machine.

Amplicon reads were quality controlled, checked for chimeras, and processed as described above. 143,066 high-quality reads were obtained and formed 1,264 OTUs at the approximate species-level (90% sequence identity). Taxonomic identity was assigned with the RDP Classifier (Wang et al., 2007) and the *dsrAB* database described by Müller et al. (2015) using a confidence threshold of 0.8.

### Statistical Analyses

Alpha diversity among samples was compared by rarefying all replicates to an even sequencing depth in Mothur v. 1.38.1 (Schloss, 2009). All other statistical analyses were performed in R, version 3.3.2 (R Core Team, 2016), without rarefaction. Only OTUs that were detected by at least three reads in three different sediment replicates were used in order to discriminate against sequencing artifacts and to enable meaningful statistical analysis. Differences in bacterial community composition were visualized using principal coordinate analysis (PCoA) plots made in the R phyloseq package 3.3.2 (McMurdie and Holmes, 2013) as based on weighted unifrac distances (Lozupone et al., 2011). Variation in community composition was tested for significance using the weighted unifrac distances and a permutational analysis of variance (PERMANOVA) in the R vegan package 2.5.1 (Oksanen et al., 2013). The package edgeR 3.16.5 (Robinson et al., 2010; McCarthy et al., 2012) was used to test for significant (FDR-corrected *p*-value < 0.05) and large (logFC ≥ 2) changes of relative OTU abundances according to both incubation time and incubation line. Corrections of the 16S rRNA gene amplicon dataset for rrn operon numbers were performed at the highest taxonomic resolution of the obtained OTUs as described before (Hausmann et al., 2016).

### Sequence Data Availability

Amplicon data of 16S rRNA genes, 16S rRNA cDNA, and *dsrB* cDNA were deposited at the Sequence Read Archive at NCBI (Leinonen et al., 2011) under the bioproject number PRJNA495895.

## Results

### Sulfate Is Readily Turned Over in Sediment Microcosms

**FIGURE 1:**
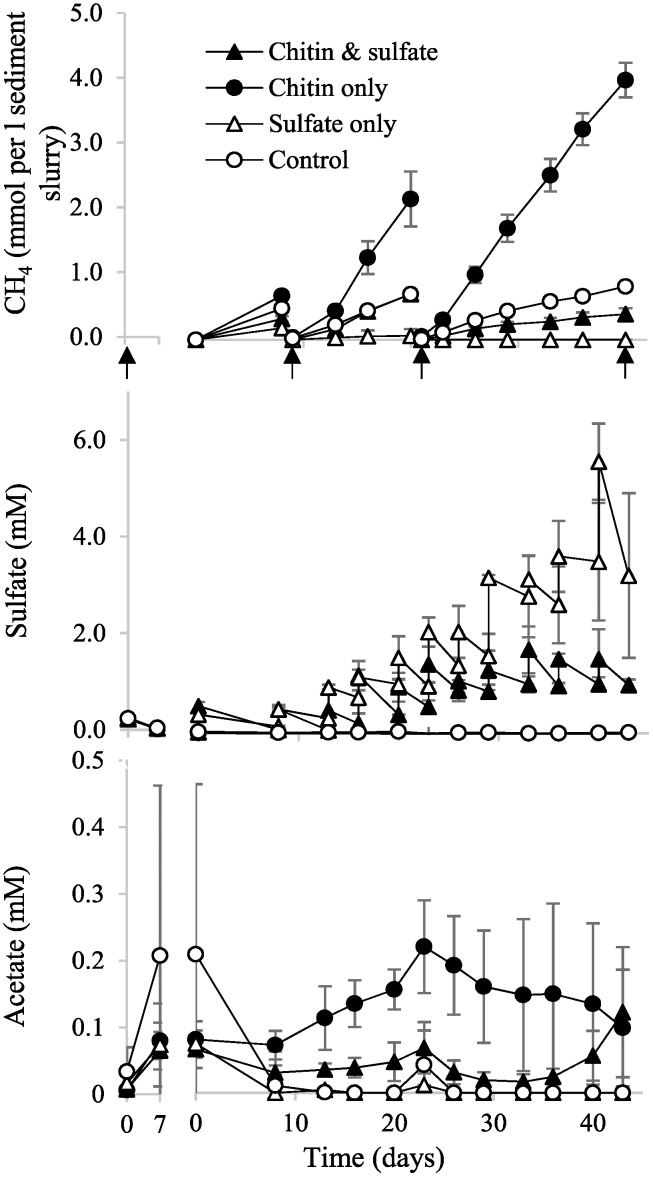
Time course of sulfate turnover and formation of metabolic end products in incubated sediment microcosms. The gap illustrates the shift from pre-incubation to the chitin and/or first sulfate amendment. Sulfate was always measured twice: before and after supplementation. Opening of microcosms under anoxic conditions for sediment sampling is indicated by black arrows. The mean and one standard deviation are shown, *n* = 3. Some error bars are smaller than the symbol size.

Twelve anoxic slurries of littoral sediment were pre-incubated for 7 days to allow for the depletion of endogenous electron donors and acceptors. Among monitored short-chained fatty acids and lactate, only acetate was detectable and accumulated transiently from 16 to 107 μM (average across all incubations). Initial sulfate concentrations were 229 ± 34 μM and depleted rapidly within the pre-incubation period to 24 μM (Figure 1), which indicated an active sulfate-reducing microbial community.

Chitin supplementation resulted in a 3.6-fold higher accumulation of CH_4_ by day 43 under methanogenic conditions as compared to the “control” (Figure 1). By contrast, in sulfate-amended treatments, CH_4_ accumulation was suppressed by 90% in the chitin-amended treatment as compared to the treatment with “chitin only” and suppressed completely in the treatment “sulfate only.” This reflects the thermodynamically more favorable degradation of organic matter by sulfate reduction (Muyzer and Stams, 2008) as compared to primary fermentation or syntrophic processes coupling to methanogenesis (Schink and Stams, 2013). Among the monitored degradation intermediates, only acetate could be detected in chitin-amended microcosms, irrespective of the presence of external sulfate. However, the standing concentrations of acetate were roughly three times higher under methanogenic conditions (142 ± 41 μM) as compared to sulfate-reducing conditions (44 ± 30 μM) and converged only at the end of the incubation toward a similar concentration of 109 μM. Parallel monitoring of CO_2_ in the headspace of all incubations showed a steady accumulation over time, which was 5.0 and 2.5 times more pronounced in the incubations “chitin only” and “chitin & sulfate” as compared to their respective controls (Supplementary Figure S2).

In all sulfate-amended treatments, sulfate turnover could be measured throughout the incubation period. In the treatment “chitin & sulfate,” sulfate turnover rates ranged between 75 and 304 nmol day^-1^ (g sediment f. wt.)^-1^. This resulted in a total of 5.3 mM sulfate being turned over. In the treatment “sulfate only,” sulfate turnover ranged from 38 to 281 nmol day^-1^ (g sediment f. wt.)^-1^ with a sudden increase to 955 nmol day^-1^ (g sediment f. wt.)^-1^ at the very end of the incubation period. This resulted in a total of 6.0 mM sulfate being turned over (Figure 1). The measured bulk sulfate turnover rates were in the range of gross SRR observed previously for the same littoral area (Bak and Pfennig, 1991a) and thus provided a good experimental framework to detect active SRM under *in situ*-like conditions.

### Sulfate Amendment Altered a Minor Portion of the Bacterial Community Structure Over Time

The initial bacterial community of the analyzed sediment was dominated by the phyla *Proteobacteria* (classes *Alpha*-, *Beta*-, *Gamma*-, and *Deltaproteobacteria*), *Bacteroidetes*, *Chloroflexi*, *Actinobacteria*, *Acidobacteria*, *Verrucomicrobia*, *Planctomycetes*, *Aminicenantes*, *Cyanobacteria/Chloroplast*, *Ignavibacteriae* or unclassified bacteria, with each of these phyla constituting more than 1% relative abundance of the detected 16S rRNA genes (Supplementary Table S1). Good’s coverage estimator (Good, 1953) of the sampled OTUs at 97% sequence identity had an average of 0.88 ± 0.02, with 90% of all samples having a coverage between 0.86 and 0.90 (5 and 95% quantiles, respectively). At a rarefied sequencing depth of 68,826 reads per replicate, the initial sediment harbored on average 14,190 ± 366 OTUs (mean ± SD), which slightly decreased to 12,701 ± 595 OTUs (mean ± SD) in the different treatments over time (Supplementary Figure S3). Total bacterial 16S rRNA gene copies stayed stable throughout a period of 21 days of incubation and decreased only slightly toward the end of the incubation time in chitin-amended treatments irrespective of sulfate addition. On average 1.4 ± 1.2 × 10^8^ 16S rRNA gene copies could be recovered per gram sediment (f. wt.) (Supplementary Figure S4).

**FIGURE 2:**
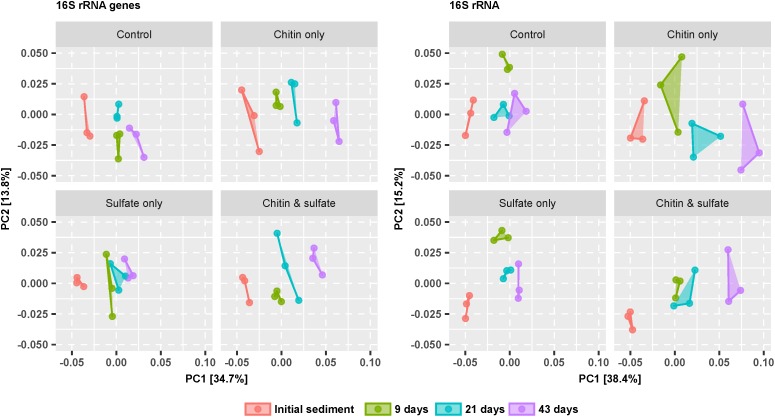
Time-resolved beta-diversity of bacterial communities in the various microcosm setups according to a principal coordinate analysis (PCoA) based on the weighted unifrac metric. Segregation of the bacterial community over time is shown for the 16S rRNA gene and 16S rRNA amplicon survey separately. Connected points of the same color represent biological replicates (*n* = 3).

The response toward treatment and over time was monitored on both the 16S rRNA gene and 16S rRNA level as proxies for changes in relative bacterial population sizes and transcriptional activities, respectively. Changes in beta diversity were visualized by a PCoA based on the weighted unifrac metric as distance measure, which takes the phylogenetic relatedness as well as the relative abundance of OTUs into account (Lozupone et al., 2011). Bacterial communities separated mainly over time and to a minor extent based on treatment, both at the 16S rRNA gene and 16S rRNA level (Figure 2). These shifts were significant (*p*-value < 0.01) as determined by a PERMANOVA analysis, where 37.5 and 39.1% of the variation in bacterial community compositions was explained by the incubation time at the level of 16S rRNA genes and 16S rRNA, respectively. Variation due to incubation type was also significant, but much smaller (12.5 and 12.4% for 16S rRNA genes and 16S rRNA, respectively). A significant interaction between treatment and incubation time indicated that bacterial communities responded differently over time depending upon the treatment, and this interaction explained 16.2 and 18.1% of the variation at the 16S rRNA gene and 16S rRNA level, respectively. When correcting the 16S rRNA gene amplicon dataset for rrn copy numbers of the respective taxa, no major differences were observed to the PCoA analysis and PERMANOVA without rrn operon correction (Supplementary Figure S5).

### *Desulfobacteraceae* and *Syntrophobacteraceae* Are the Dominant SRM Families in Initial Lake Constance Sediment

**FIGURE 3:**
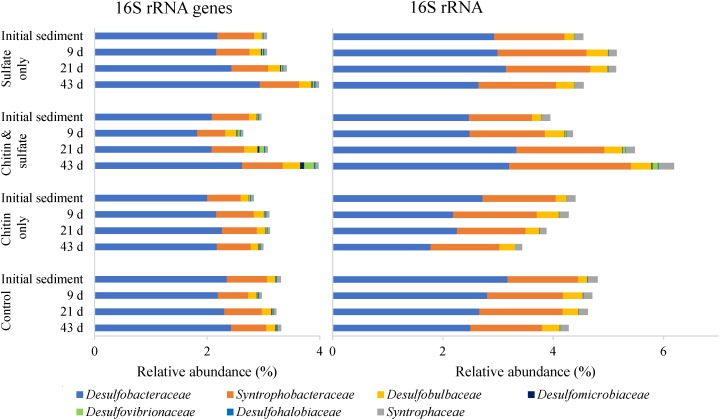
Community composition of recognized SRM families in the initial sediment and over time in the various microcosm setups. For *Syntrophaceae*, only members of the genera *Desulfobacca* and *Desulfomonile* were considered. Bars represent the mean relative abundance for each treatment and time point (*n* = 3).

In the initial sediment, relative abundances of recognized SRM lineages accounted for 3.14 ± 0.04 and 4.30 ± 0.06% of all 16S rRNA gene and 16S rRNA sequences, respectively, and were comprised of seven different families, all affiliated to the *Deltaproteobacteria* (Figure 3). Both types of sequencing analysis revealed *Desulfobacteraceae* (207 OTUs) and *Syntrophobacteraceae* (86 OTUs) as the most abundant and transcriptionally active among all recognized SRM families in the initial sediment. Members of the *Desulfobulbaceae* (34 OTUs), *Desulfomicrobiaceae* (3 OTUs), *Desulfovibrionaceae* (22 OTUs), *Desulfohalobiaceae* (2 OTUs), and *Syntrophaceae* (31 OTUs; only *Desulfobacca*- and *Desulfomonile*-related) represented in sum only 7.7 and 7.8% of 16S rRNA gene and 16S rRNA sequences related to recognized SRM, respectively (Supplementary Table S2). Among the ten most abundant SRM-related OTUs, seven had a relative abundance of >0.1%, which is typically taken as a cutoff to delineate abundant microorganisms from the rare biosphere (Lynch and Neufeld, 2015). In particular, those related to the dominating families *Desulfobacteraceae* and *Syntrophobacteraceae* accounted for 60.7 and 63.2% of all 16S rRNA gene and 16S rRNA sequences affiliated with known SRM (Supplementary Table S2).

Over time, the 16S rRNA gene-based community composition of recognized SRM changed only to a small extent in sulfate-amended treatments and stayed largely stable in the methanogenic controls, irrespective of chitin amendment (Figure 3). Sulfate amendment in general stimulated increases in *Desulfobacteraceae* (from 2.13 to 2.79%) and *Desulfobulbaceae* (from 0.14 to 0.26%) 16S rRNA gene sequences. On the contrary, the relative abundance of *Desulfovibrionaceae* increased only in the “chitin & sulfate” treatment (from 0.01 to 0.18%). All other recognized SRM families showed no pronounced changes.

Responses toward the individual treatments were more pronounced at the 16S rRNA level (Figure 3). While the relative amount of *Desulfobacteraceae* 16S rRNAs increased again under “chitin & sulfate” (from 2.46 to 3.18%), they first increased and then dropped in the treatment “sulfate only” and clearly declined in both methanogenic treatments. On the contrary, *Desulfobulbaceae* 16S rRNAs increased in all treatments, irrespective of chitin, and sulfate supply. Interestingly, *Syntrophobacteraceae* revealed the most pronounced change, with their relative 16S rRNA numbers being doubled in the treatment “chitin & sulfate” until the end of the incubation time (from 1.17 to 2.19%) but staying largely stable in all other treatments. A similar trend was observed for *Desulfovibrionaceae*, *Desulfomicrobiaceae*, and *Desulfohalobiaceae.*

At the level of individual OTUs, 119 and 73 of a total of 397 SRM-related OTUs showed a steady increase (*R*^2^ ≥ 0.80) within 21 days of incubation at the 16S rRNA level in the “chitin & sulfate” and “sulfate only” treatments, respectively (Supplementary Table S2). For example, these included the dominating *Desulfobacteraceae* OTU10 and *Syntrophobacteraceae* OTU30 in the “chitin & sulfate” treatment. At the 16S rRNA gene level, responses were less pronounced with 84 and 88 SRM-related OTUs that showed a steady increase in the “chitin & sulfate” and “sulfate only” treatments, respectively. Large relative abundance changes among OTUs (logFC > 2.0; FDR-corrected *p*-value < 0.05) were restricted to a small subset of OTUs steadily increasing in their relative 16S rRNA or 16S rRNA gene copies. The majority of these OTUs belonged to the rare biosphere (<0.1% relative abundance) in the initial sediment and affiliated mainly to the *Desulfovibrionaceae* and *Desulfobacteraceae* (Supplementary Table S3).

### *Desulfobacteraceae* and *Syntrophobacteraceae* Dominate dsrB-Transcribing Microorganisms

The 16S rRNA (gene)-based analysis was supported by screening for microorganisms actively transcribing their *dsrB* as a functional marker for SRM (Müller et al., 2015). Good’s coverage estimator (Good, 1953) of the sampled *dsrB* cDNA OTUs in the initial sediment had an average of 0.93 ± 0.05 with 90% of all samples having a coverage between 0.82 and 0.97 (5 and 95% quantiles, respectively). Consistent with the data of the bacterial 16S rRNA gene and 16S rRNA analyses, *Desulfobacteraceae* and *Syntrophobacteraceae* were the most abundant families detected by *dsrB* cDNA amplicon sequencing (Table 1). Almost half of the *dsrB*-transcribing microorganisms were represented by these two families in the initial sediment, of which the most abundant *dsrB* OTU1 (*Syntrophobacteraceae*) and *dsrB* OTU2 (*Desulfobacteraceae*) accounted for 12.3 and 11.6% of all *dsrB* transcripts, respectively (Supplementary Table S4). In addition, results of the initial community of Lake Constance sediment revealed that most of the 14 detected *dsrB*-transcribing family lineages were affiliated with the *Deltaproteobacteria* supercluster (93.96% of all sequences, Table 1). Only 1.64 and 0.09% affiliated to the *Firmicutes* group and the *Acidobacteria* [formerly environmental supercluster, (Hausmann et al., 2018)], respectively. Among uncultured family-level lineages, lineage 6 and 5 (both *Firmicutes* group) were the most dominant ones and represented 1.36 and 0.24% of the *dsrB*-transcribing community.

**Table 1:** Overview of the *dsrB*-transcribing community in initial littoral sediment of Lake Constance.

Reductive bacterial type *DsrAB*	Family	Relative abundance (%)	Number of OTUs
*Deltaproteobacteria* supercluster	*Desulfobacteraceae*	23.44 ± 1.03	39
	*Syntrophobacteraceae*	22.36 ± 0.56	40
	*Desulfobacca acetoxidans* lineage	12.46 ± 0.79	49
	*Desulfobulbaceae*	8.99 ± 0.96	28
	*Thermodesulfobacteria*	2.4 ± 0.18	3
	*Desulfonatronumaceae*	1.49 ± 0.06	13
	LA-*dsrAB Firmicutes*	0.39 ± 0.05	10
	*Desulfovibrionaceae*	0.24 ± 0.03	4
	Uncultured family-level lineage 11	0.22 ± 0.05	4
	*Desulfomicrobiaceae*	0.03 ± 0.01	2
	*Desulfomonile tiedjei* lineage	0.02 ± 0.02	1
	Unclassified	21.92 ± 0.78	138
Environmental supercluster /	Uncultured family-level lineage 8	0.05 ± 0.01	2
*Acidobacteria*	Uncultured family-level lineage 9	0.04 ± 0.02	2
*Firmicutes* group	Uncultured family-level lineage 6	1.36 ± 0.1	8
	Uncultured family-level lineage 5	0.24 ± 0.04	15
	Unclassified	0.04 ± 0.01	3
Unclassified	Unclassified	4.3 ± 0.43	72

*Classification was performed according to Müller et al. (2015). The mean and one standard deviation of the relative abundance are given for n = 12.*

**FIGURE 4:**
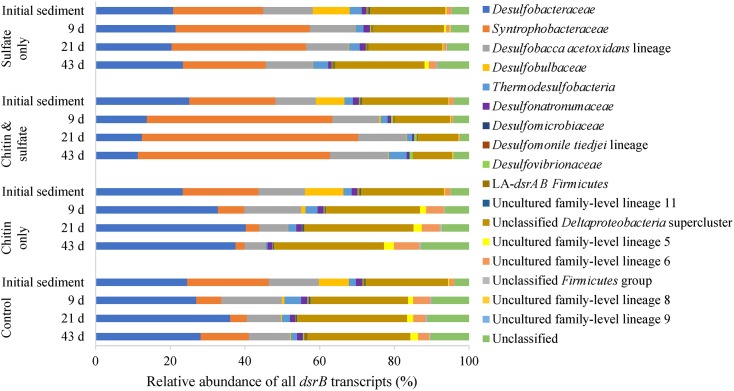
Relative abundance of *dsrB* transcripts affiliated to *dsrAB* family-level lineages according to Müller et al. (2015). Bars represent the mean relative abundance of each lineage for the respective treatment and time point (*n* = 3).

Similar to the results of the 16S rRNA analysis, the amendment of chitin and sulfate led to a strong increase in the relative abundance of *Syntrophobacteraceae dsrB* transcripts, which more than doubled from 23.1 to 57.9% after 21 days (Figure 4). Also in the treatment “sulfate only,” the relative abundance of *Syntrophobacteraceae dsrB* transcripts increased transiently from 24.2 to 36.1% during the first 21 days but declined thereafter again toward 22.2%. On the contrary and in disagreement with the 16S rRNA analysis, *Desulfobacteraceae dsrB* transcripts strongly decreased in relative abundance in the treatment “chitin & sulfate.” The opposite trend was observed in the methanogenic “chitin only” and “control” treatments, where the relative numbers of *Syntrophobacteraceae dsrB* transcripts decreased and *Desulfobacteraceae dsrB* transcripts increased. Increased relative abundances of transcribed *dsrB* were also observed for minor *dsrB* transcribing lineages. These comprised members of the *Desulfomicrobiaceae*, *Desulfovibrionaceae*, *Thermodesulfobacteria* and *Desulfobacca acetoxidans*-lineage, with the increase in relative *dsrB* transcript numbers being more pronounced in the treatment “chitin & sulfate” than in “sulfate only” (Figure 4).

The OTU composition of *dsrB*-transcribing microorganisms was dominated by 16 *dsrB* OTUs, each representing more than 1% of *dsrB* transcripts and accounting together for 62.3% of the total relative abundance of *dsrB* transcripts in the initial sediment (Supplementary Table S4). In particular, these *dsrB* OTUs were affiliated to the *Desulfobacteraceae* (OTU2, OTU5), *Syntrophobacteraceae* (OTU1, OTU7, OTU8), *Desulfobacca acetoxidans* lineage (OTU4, OTU10, OTU11), *Desulfobulbaceae* (OTU12, OTU14, OTU27), *Thermodesulfobacteria* (OTU6) and unclassified *Deltaproteobacteria* (OTU3, OTU13, OTU15, OTU19). Among these highly transcriptionally active OTUs, *dsrB* OTU1 (*Syntrophobacteraceae*) was outstanding since it increased more than threefold in relative *dsrB* transcript numbers in the “chitin & sulfate” treatment where it comprised 39% of all detected transcripts at the end of the incubation. Similarly, *dsrB* OTU1 comprised 26% of all *dsrB* transcripts at day 21 in the treatment “sulfate only” and declined thereafter to a level similar to the initial sediment (15% of all *dsrB* transcripts). In contrast, *dsrB* OTU1 strongly declined in the methanogenic “chitin only” and “control” treatments, which was counterbalanced by a strong increase in *dsrB* transcripts of *Desulfobacteraceae dsrB* OTU2 (up to 26% of *dsrB* transcripts).

## Discussion

Sulfate reduction in littoral sediments of Lake Constance was estimated to contribute about 25% to the anaerobic degradation of organic matter during its eutrophic phase in the second half of last century (Bak, 1988). In comparison to oligotrophic lakes, the respective SRR of up to 20 mmol sulfate m^-2^ d^-1^ are in the upper range but are comparable to rates observed in other mesotrophic and eutrophic lakes (Holmer and Storkholm, 2001). This importance of sulfate reduction for limnetic sedimentary processes is reflected in the pronounced relative abundance of SRM (3% of all bacterial 16S rRNA gene sequences, Figure 3), even in the currently oligotrophic status of the lake. When normalized against the ca. 10^8^ 16S rRNA gene copies of the total microbial community per gram sediment (f. wt.) (Supplementary Figure S4) and taking into account the varying amount of rrn operons (1–21) on the genomes of different microbial species (Stoddard et al., 2015) and the dominance of bacteria over archaea (Schwarz et al., 2007), this would result in roughly 10^5^–10^6^ SRM cells per gram sediment. Our results compare well to cultivation-based abundance estimates of SRM in littoral sediments of Lake Constance, which detected 6 × 10^6^ cultivable cells per ml of sediment (Bak and Pfennig, 1991b). In addition, they correspond well to 16S rRNA gene-based estimates of SRM in sediments of other lakes, such as eutrophic Lake Vechten (ca. 3%, Diao et al., 2018), mesotrophic Lake Kizaki (ca. 4%, Li et al., 1999), meso-eutrophic Lake Kinneret (5–9%, Schwarz et al., 2007), or several water reservoirs of oligotrophic to eutrophic status (ca. 3–5%, Wobus et al., 2003).

The difficulty in delineating active SRM in environmental surveys is their polyphyletic distribution in the tree of life (Rabus et al., 2013) and the fact that many putative SRM are only known by their *dsrA*/*B* sequences (Müller et al., 2015) or their *dsrAB*-carrying metagenome-assembled genomes (Anantharaman et al., 2018; Hausmann et al., 2018; Zecchin et al., 2018). To address this methodological problem, we used a tripartite amplicon sequencing approach of 16S rRNA genes, 16S rRNA, and *dsrB* transcripts as proxies for relative population abundance, general transcriptional activity, and activation of the sulfate reduction pathway, respectively. This tripartite approach was applied to lake sediment microcosms that actively consumed sulfate in the presence or absence of chitin as an abundant aquatic polysaccharide (Gooday, 1990) and general substrate for the whole organic carbon degradation network. All three amplicon approaches jointly identified members of the *Desulfobacteraceae* and *Syntrophobacteraceae* as the dominant SRM populations in the initial littoral sediment of Lake Constance. Both SRM groups not only dominated in terms of relative abundance but also in their general and *dsrB* transcriptional activity among both recognized SRM and in comparison to uncultured *dsrAB* family-level lineages (Figure 3, 4). This was corroborated in the time course analyses of sulfate-consuming sediment microcosms irrespective of chitin amendment. Here, *Desulfobacteraceae* showed clear population and general transcriptional increases whereas *Syntrophobacteraceae* mainly responded by increasing their transcriptional activity (Figure 3). The latter was clearly evident not only at the level of the 16S rRNA but also by the respective *dsrB* transcripts. Especially one OTU, *Syntrophobacteraceae-*affiliated *dsrB* OTU1, dominated relative *dsrB* transcript dynamics in sediment microcosms irrespective of chitin amendment. It constituted either up to 26–40% of *dsrB* transcripts in sulfate-amended or less than 1% in methanogenic microcosms toward the end of incubation (Supplementary Table S4). It is very likely that these strong shifts in one OTU affected relative *dsrB* transcript dynamics of other OTUs and lineages, which therefore have to be interpreted with caution. This is especially true for a functional marker gene, where transcriptional dynamics in a few strongly responding members are not stabilized by a majority of non-responding members as is often the case in 16S rRNA amplicon surveys.

Our results compare well to studies, which analyzed the sulfate-reducing community of lake sediments at the level of 16S rRNA genes only. As in littoral sediment of Lake Constance, members of the *Desulfobacteraceae* constituted the largest fraction of sediment SRM in lakes worldwide including Lake Geneva in Switzerland (Haller et al., 2011), Lake Biwa and Oko in Japan (Watanabe et al., 2013), Lake Vechten in the Netherlands (Diao et al., 2018), and Lake Kinneret in Isreal (Schwarz et al., 2007). While in Lake Constance and Lake Kinneret, *Syntrophobacteraceae* were the second-most dominant SRM group (this study, Schwarz et al., 2007), this position was taken by *Desulfobulbaceae* in other lakes (Watanabe et al., 2013; Diao et al., 2018). *Desulfobulbaceae* were also detected in initial littoral sediment of Lake Constance, constituting the third most abundant SRM group (Figure 3) and being transcriptionally active at the 16S rRNA and *dsrB* level (Figure 3, 4). Placing the *Desulfobulbaceae dsrB* and 16S rRNA sequences into the respective phylogenetic trees revealed that these OTUs were more closely related to classical SRM such as members of the genera *Desulfobulbus* and *Desulfocapsa* than to the recently described sulfide-oxidizing cable bacteria of the genera *Electrothrix* and *Electronema* (Trojan et al., 2016).

From a physiological perspective, cultured representatives of the *Desulfobacteraceae* and *Syntrophobacteraceae* are known to be metabolically versatile sulfate reducers with most members being chemoorganotrophs that oxidize organic matter completely to CO_2_ (Kuever, 2014a,c). A fermentative lifestyle in the absence of sulfate is described for members of both families, with members of the *Syntrophobacteraceae* being known to form syntrophic associations with H_2_ or formate-utilizing partner microorganisms under such conditions (Kuever, 2014a,c). Therefore, they seem to be well adapted to freshwater environments characterized by a low sulfate content and steeply declining gradients of sulfate downward the sediment (e.g., Bak and Pfennig, 1991a). In addition, *Desulfobulbaceae* accounted for the core SRM community in the initial sediment. In contrast to *Desulfobacteraceae* and *Syntrophobacteraceae*, members of this family are typically incomplete oxidizers of organic compounds to acetate and CO_2_ (Kuever, 2014b). In the absence of sulfate, several members of the *Desulfobulbaceae* are reported to use nitrate as a terminal electron acceptor (Kuever, 2014b), which may hint toward a different range of their ecological niche as compared to *Desulfobacteraceae* and *Syntrophobacteraceae*. In addition, a few members of the *Desulfobulbaceae* are even unable to use sulfate as terminal electron acceptor, but use thiosulfate, sulfur or polysulfide instead or conserve energy by disproportionation of sulfur compounds (Kuever, 2014b).

An important aspect of our study was that we screened not only for the presence and activity of recognized SRM lineages but also included uncultured *dsrAB* lineages of putatively novel SRM into our analysis. The used primer mixes DSR1728F-mixA and DSR4R-mix cover 89% of the known sequence space of the phylogenetic branch of bacterial, reductively operating *dsrAB* when allowing for one weighted mismatch (Müller et al., 2015). It was surprising to observe that uncultured, family-level *dsrAB* lineages accounted for only 1.9% of all *dsrB* transcripts in the initial sediment, with uncultured lineage 5 and 6 representing the most transcriptionally active ones (Table 1). This was in contrast to freshwater wetlands, which constitute important low-sulfate habitats for SRM as well. Here, typically uncultured lineages constitute a large fraction of the *dsrAB* diversity (Pester et al., 2012). One explanation could be that many of the wetlands analyzed so far comprise acidic peatlands. The *dsrAB*-carrying community in these habitats is dominated by members of uncultured lineage 8 (Loy et al., 2004; Steger et al., 2011; Pester et al., 2012; Pelikan et al., 2016), which were recently described as *Acidobacteria* encoding the complete pathway of dissimilatory sulfate reduction (Anantharaman et al., 2018; Hausmann et al., 2018).

## Conclusion

Lake Constance is one of best studied lakes worldwide and among the most important long-term ecological research sites in limnology with data records over a hundred years period (Güde and Straile, 2016). Its sediment microbiota has been extensively studied over the past decades (see work by Norbert Pfennig, Ralf Conrad, Meinhard Simon, Bernhard Schink), yet a detailed characterization of its SRM was missing. Our study is the first systematic approach to quantify and characterize SRM including uncultured *dsrAB* lineages not only in Lake Constance, but for lake sediments in general. Our results clearly demonstrated that members of the *Desulfobacteraceae* and *Syntrophobacteraceae* dominate the SRM sediment community in both abundance and transcriptional activity with uncultured *dsrAB* lineages contributing only a minor proportion to the overall sulfate-reducing microbial community. Future research needs to establish the individual ecological niches of the different SRM lineages identified.

## Author Contributions

SW designed and performed the experiments, analyzed the data, and wrote the manuscript. MP designed the experiments and wrote the manuscript.

## Conflict of Interest Statement

The authors declare that the research was conducted in the absence of any commercial or financial relationships that could be construed as a potential conflict of interest.

## Supplementary Material

FIGURE S1 Overview of the experimental set up. Time points of sulfate and substrate amendment are shown by blue and green bars, respectively. At the same days when sulfate was amended, all replicates were sampled for gasses (CH_4_ and CO_2_), sulfate and turnover intermediates. Initial sediment samples were taken directly at the onset of the experiment. Sediment samples for nucleic acids extraction were removed after 9, 21, and 43 days of initial chitin amendment (indicated by black color).

FIGURE S2 Overview of the accumulated CO_2_ in the headspace of the different microcosm setups. The gap illustrates the shift from pre-incubation to the chitin and/or first sulfate amendment. The mean and one standard deviation are given, *n* = 3. Some error bars are smaller than the symbol size.

FIGURE S3 Time-resolved changes in the number of observed bacterial species-level OTUs in the individual treatments (16S rRNA gene analysis, 97% identity) when rarefied to an even sequencing depth of 68,826 reads per replicate (*n* = 3).

FIGURE S4 Abundance of total bacterial and archaeal 16S rRNA genes per gram sediment (fresh weight) in the various microcosm setups as revealed by qPCR. The mean and one standard deviation are given, *n* = 3. Some error bars are smaller than the symbol size.

FIGURE S5 Time-resolved beta-diversity of bacterial communities in the various microcosm setups according to a PCoA based on the weighted unifrac metric. Segregation of the bacterial community over time is shown for the relative abundance of 16S rRNA gene OTUs (97% sequence identity) when corrected for the respective rrn copy number of the represented taxa at the highest possible taxonomic resolution. Connected points of the same color represent biological replicates (*n* = 3).

TABLE S1 Overview of the total bacterial community at the phylum level (for *Proteobacteria*, class level) in initial littoral sediment of Lake Constance (Supplementary Table S1a) and the individual OTU level for each replicate and time point (Supplementary Table S1b).

TABLE S2 Summary of all 16S rRNA (gene) OTUs at the approximate species-level (97% identity), which were affiliated to recognized lineages of sulfate-reducing microorganisms. For each OTU, the phylogenetic affiliation, the mean relative abundance at the 16S rRNA gene and 16S rRNA level in the initial sediment, and the change in relative abundance at the 16S rRNA gene and 16S rRNA level in the various treatments (provided as slope of a linear regression and the respective *R*^2^-value) is given.

TABLE S3 Overview of strongly positive (logFC > 2) and significant (FDR-corrected *p*-value < 0.05) relative abundance changes among 16S rRNA (gene) OTUs affiliated to recognized SRM lineages in the treatments “chitin & sulfate” or “sulfate only” as compared to the initial community and the respective methanogenic treatments over time.

TABLE S4 Summary of *dsrB* transcript OTUs at the approximate species-level (90% nucleotide sequence identity), which were affiliated to the phylogenetic branch of reductively operating *dsrAB*. For each OTU, the phylogenetic affiliation, the mean relative transcript abundance in the initial sediment, and the change in relative transcript abundance in the various treatments (provided as slope of a linear regression and the respective *R*^2^-value) is given in Supplementary Table S4a. Relative abundances of each *dsrB* transcript OTU per time point and replicate are given in Supplementary Table S4b.
