# Supplementary figures and images for: The Active Sulfate-Reducing Microbial Community in Littoral Sediment of Oligotrophic Lake Constance
Source: Front Microbiol. 2019 Feb 13;10:247. doi: 10.3389/fmicb.2019.00247 (PMC6381063; doi:10.3389/fmicb.2019.00247)

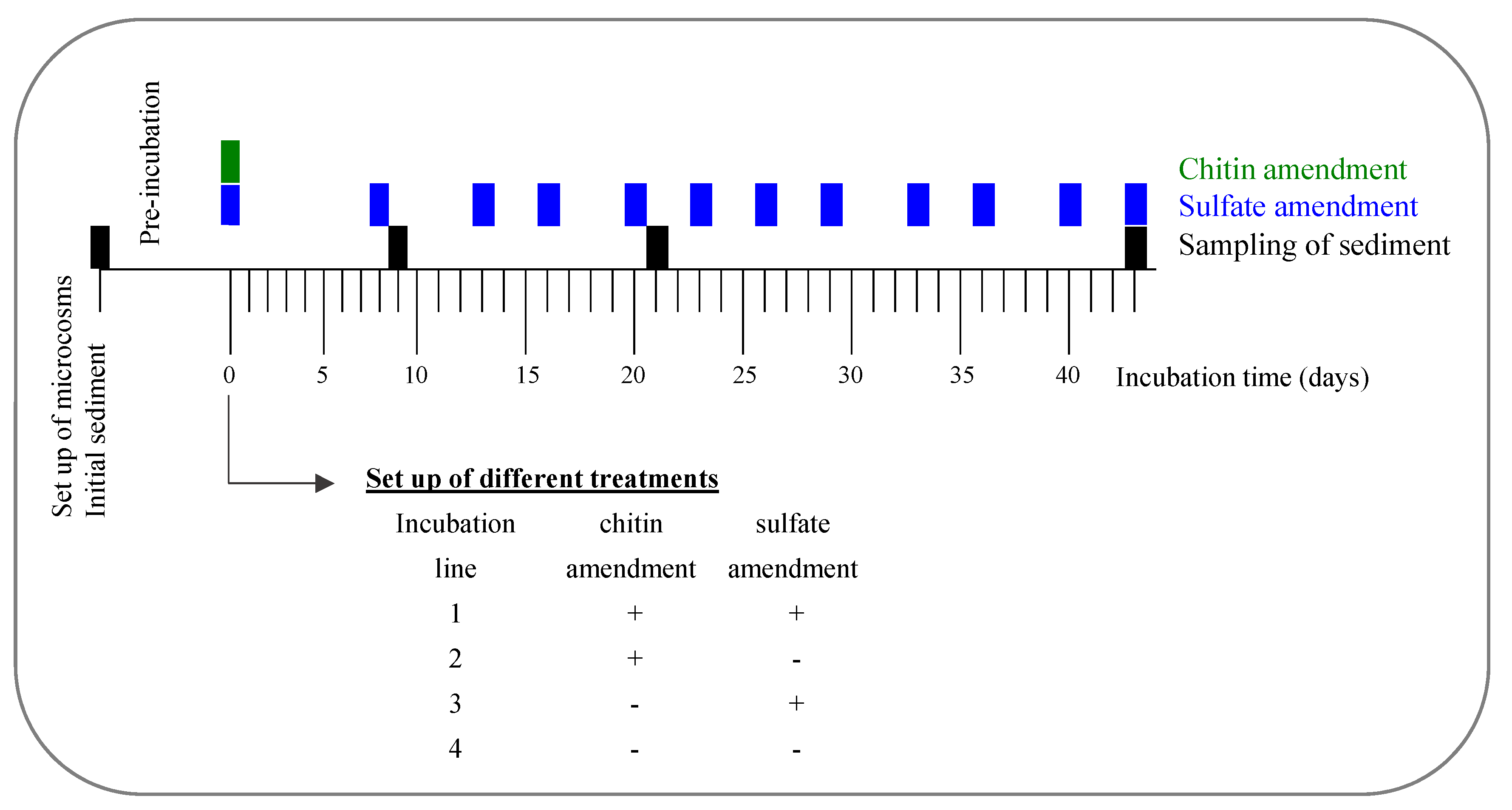

Supplement: FIGURE S1 — Overview of the experimental set up. Time points of sulfate and substrate amendment are shown by blue and green bars, respectively. At the same days when sulfate was amended, all replicates were sampled for gasses (CH4 and CO2), sulfate and turnover intermediates. Initial sediment samples were taken directly at the onset of the experiment. Sediment samples for nucleic acids extraction were removed after 9, 21, and 43 days of initial chitin amendment (indicated by black color). [file Image_1.TIF]

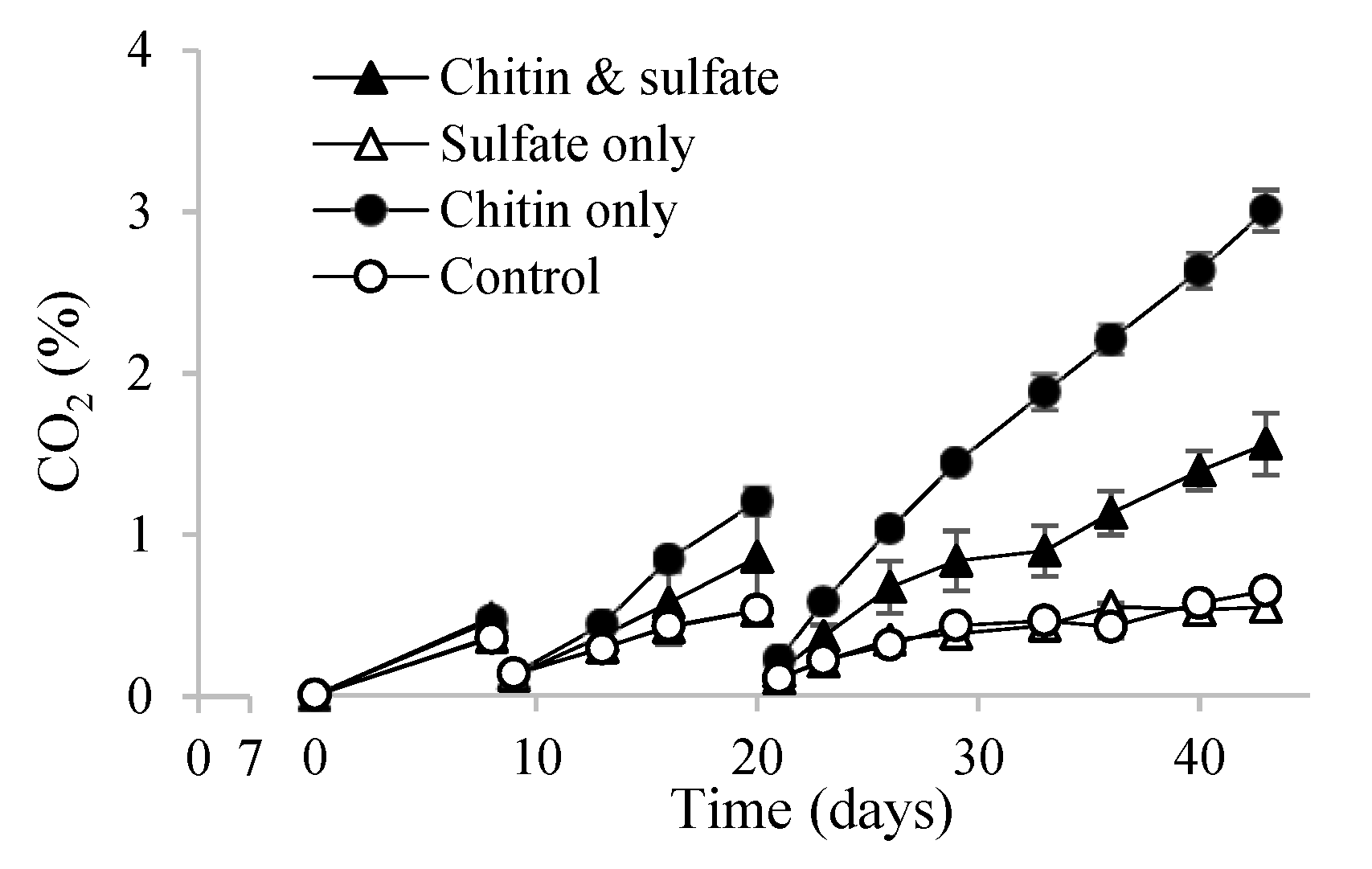

Supplement: FIGURE S2 — Overview of the accumulated CO2 in the headspace of the different microcosm setups. The gap illustrates the shift from pre-incubation to the chitin and/or first sulfate amendment. The mean and one standard deviation are given, n = 3. Some error bars are smaller than the symbol size. [file Image_2.TIF]

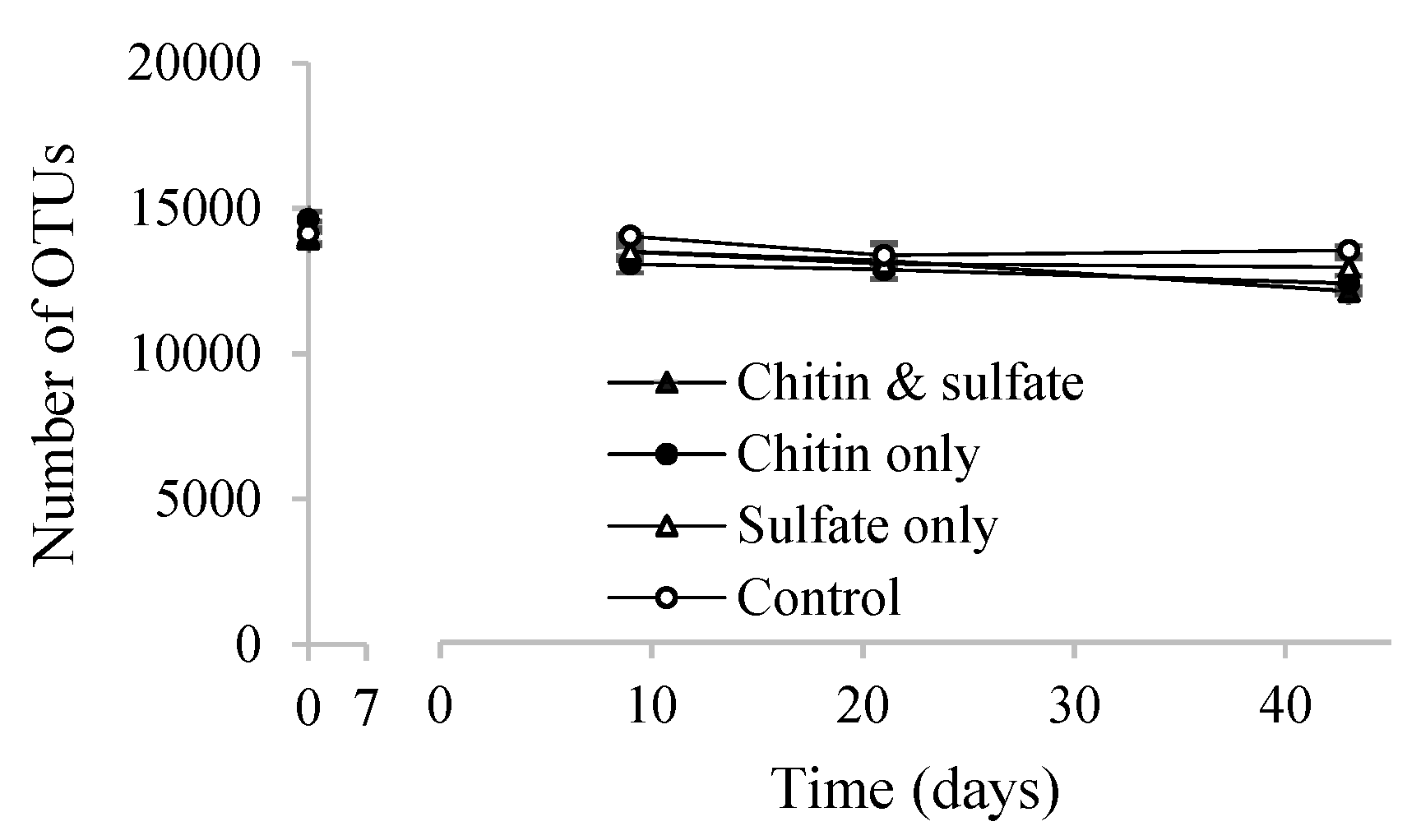

Supplement: FIGURE S3 — Time-resolved changes in the number of observed bacterial species-level OTUs in the individual treatments (16S rRNA gene analysis, 97% identity) when rarefied to an even sequencing depth of 68,826 reads per replicate (n = 3). [file Image_3.TIF]

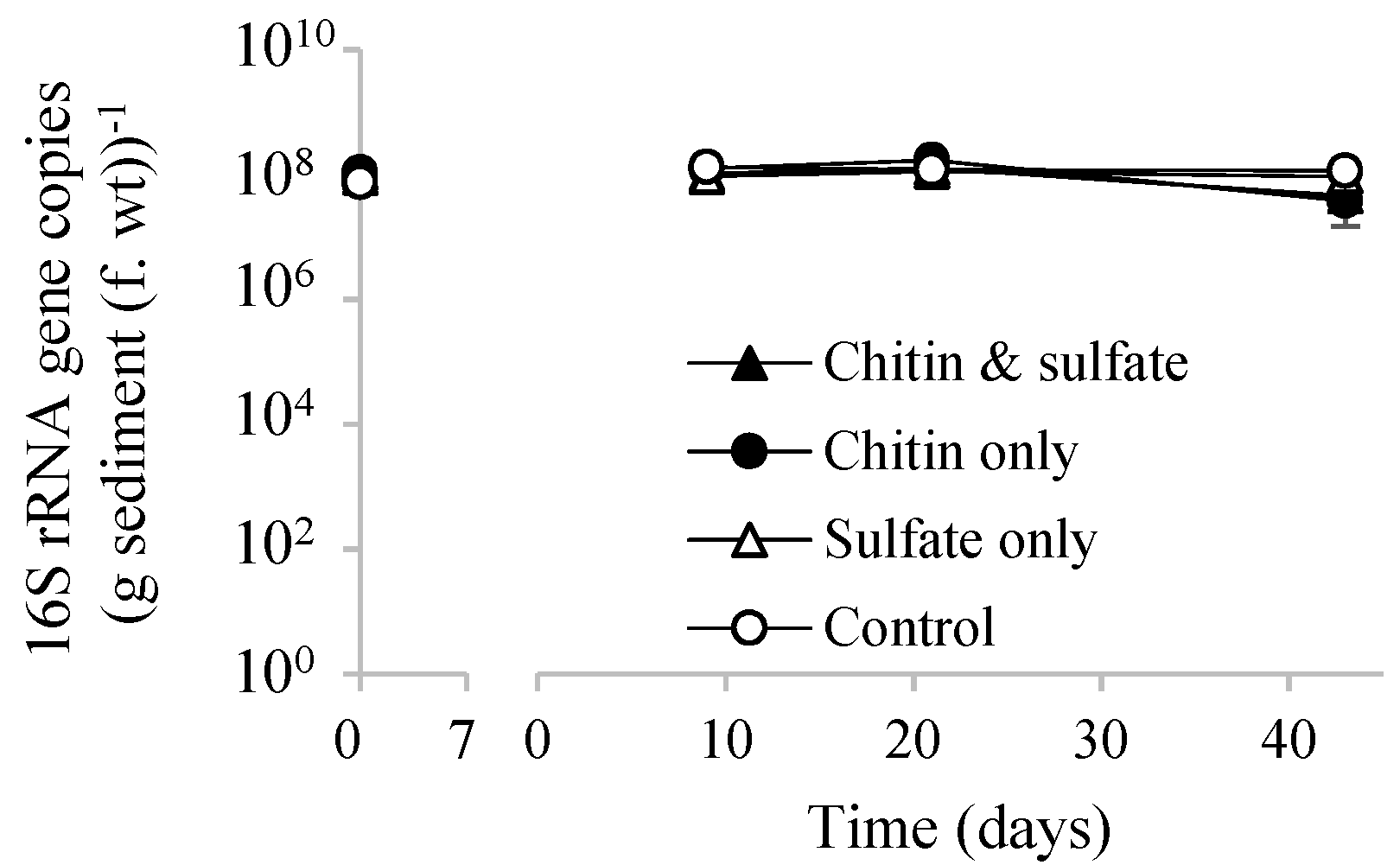

Supplement: FIGURE S4 — Abundance of total bacterial and archaeal 16S rRNA genes per gram sediment (fresh weight) in the various microcosm setups as revealed by qPCR. The mean and one standard deviation are given, n = 3. Some error bars are smaller than the symbol size. [file Image_4.TIF]

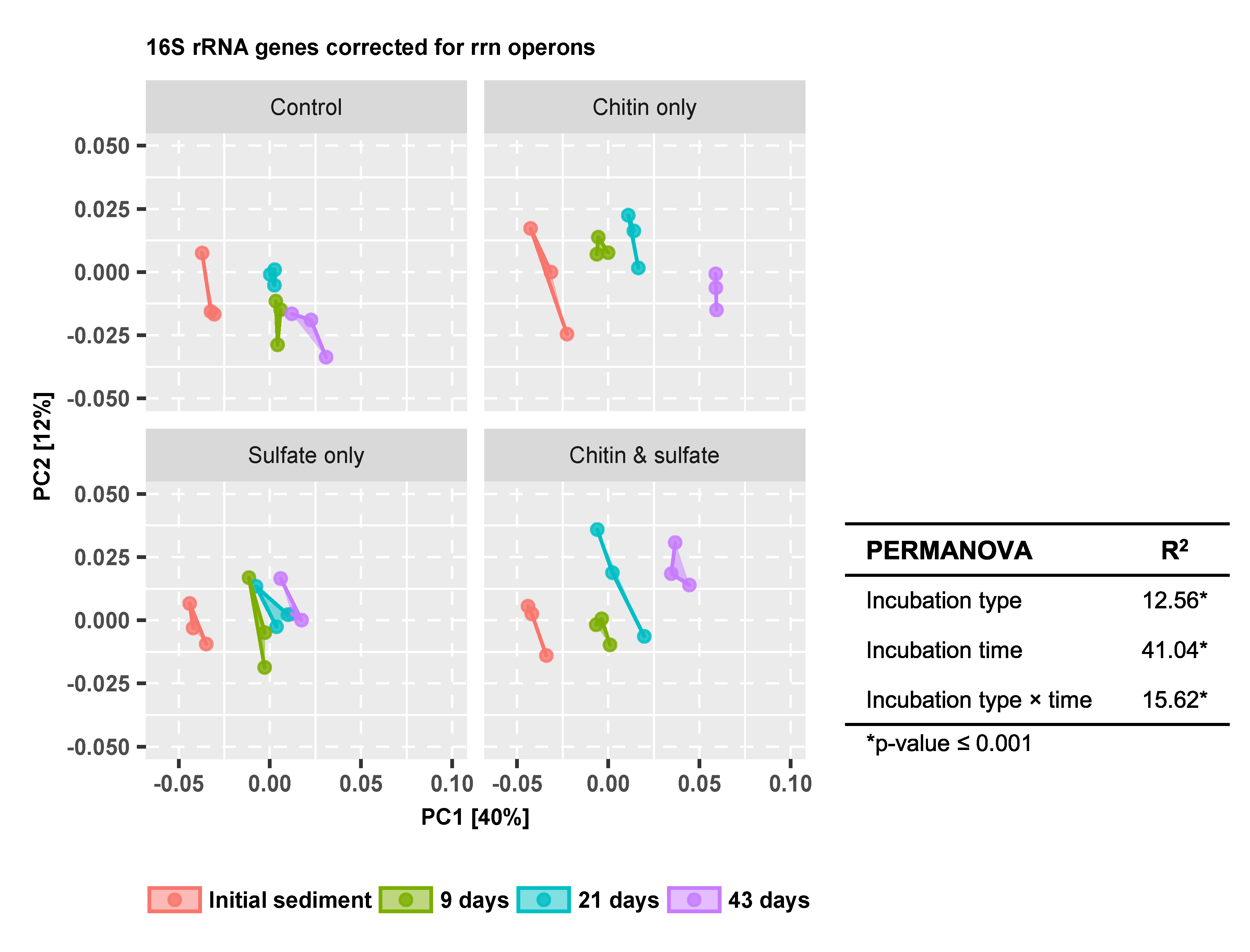

Supplement: FIGURE S5 — Time-resolved beta-diversity of bacterial communities in the various microcosm setups according to a PCoA based on the weighted unifrac metric. Segregation of the bacterial community over time is shown for the relative abundance of 16S rRNA gene OTUs (97% sequence identity) when corrected for the respective rrn copy number of the represented taxa at the highest possible taxonomic resolution. Connected points of the same color represent biological replicates (n = 3). [file Image_5.TIFF]
